# Sickness absence and sickness presence in relation to office type: An observational study of employer-recorded and self-reported data from Sweden

**DOI:** 10.1371/journal.pone.0231934

**Published:** 2020-04-29

**Authors:** Loretta G. Platts, Aram Seddigh, Erik Berntson, Hugo Westerlund

**Affiliations:** 1 Department of Psychology, Stress Research Institute, Stockholm University, Stockholm, Sweden; 2 Department of Psychology, Stockholm University, Stockholm, Sweden; TED University, TURKEY

## Abstract

**Objectives:**

Previous research suggesting that open-plan office environments are associated with higher rates of sickness absence rely on self-reports which can be affected by recall bias. This paper investigates the associations of sickness absence, obtained from employer records as well as self-reports, with office type (cell offices and different sizes of open-plan offices). It additionally studies whether office type is associated with sickness presence.

**Methods:**

Employees from two private and one public sector organization were recruited to the study. Office type was ascertained by direct observation or from employee responses to an online survey. Control variables were gender, age, public/private sector and education level. Number of days and episodes of sickness absence were calculated from employer absence records and regressed on office type using negative binomial regression (n = 988). Self-reports of sickness absence and presence were regressed on office type using ordered logistic regression (n = 1237).

**Results:**

Office type was generally not associated with employer records of number of episodes or days of sickness absence, except that the total number of days of leave was higher in flex offices compared to cell offices (IRR = 2.46, p = 0.007). In general, office type was not associated with self-reported days of sickness absence, apart from participants working in medium-sized open-plan offices who had 0.42 higher log-odds of absence than those working in cell offices (p = 0.004). Office type was not associated with self-reported sickness presence.

**Conclusions:**

Office type was not associated with sickness presence nor, in general, with sickness absence, whether obtained from self-reports or company records. It is not possible to conclude from this study that open-plan offices are associated with greater sickness absence or sickness presence compared to cell offices.

## 1. Introduction

A major trend in organizations worldwide is the conversion of office space from cell offices to various sorts of open-plan office environments and activity-based flexible offices in which employees do not have an individually assigned desk. [1] By taking up less space and using space more flexibly, open-plan and flex offices potentially offer cost savings to employers and provide opportunities for different modes of working that may suit employees. However, research into the implications for employee health and well-being of this aspect of the work environment is still quite limited. [2]

A recent systematic review into office design which included a wide range of health and psychological outcomes found only fifteen relevant studies. [3] The authors concluded that, compared with individual offices, employees’ health was poorer in shared or open-plan offices. Prior research has associated office type with health outcomes such as the common cold, suggesting the importance of open-plan designs in exposing white-collar workers to infectious agents, thereby affecting their vulnerability to ill health. [4] Another mechanism may be that office design has been associated with factors such as distraction, cognitive stress and dissatisfaction with the environment, perhaps because employees working in open-plan offices have limited personal control over their environment, such as regulating temperature and noise levels. [5–8] This may limit employees’ adjustment latitude and increase rates of illness. [9–11]

Illness may affect work by causing the employee to be absent from work or to attend work while ill, the latter described as sickness presence or presenteeism. Both sickness absence and presence are common and carry substantial economic and social costs, not least to employers. [12] Sickness absence and presenteeism can be understood as resulting from a two-step decision process in which an employee feels ill and decides whether to attend work that day. [13] Consequently both phenomena correlate with personal and work-related factors that affect vulnerability to illness, with higher levels of absence and presenteeism correlating with high psychosocial stress and poor social support. [14,15] Whether an ill employee decides to attend work is influenced by a second set of factors, such as whether the employee can reduce or alter their work effort (adjustment latitude) and the likely negative consequences of absence (attendance requirements), which relate to the employee’s attitudes and personal situation as well as organizational policies and nature of work tasks [13,16–18].

To our knowledge, only three studies have examined the associations between office type and sickness absence. [19–21] The characteristics of these studies and their results are summarized in Table 1. The largest study, of Danish employees, found that participants working in open-plan offices containing at least six people reported 62% more days of sickness absence compared to employees in cell offices, while employees sharing offices with 1–6 people reported intermediate levels of sickness absence. [19] In contrast, neither of the Swedish studies managed to show a relationship between working in an open office and length of sickness absence. [20,21] Another dimension of sickness absence is number of episodes of sickness absence. In relation to this measure, one study showed that employees in small, medium and large open-plan offices were more likely to report two or more short sickness absence spells of one week or less. [20] Both Swedish studies reported associations for flex offices and episodes of sickness absence, but these were inconsistent. The earlier study reported that compared to those working in cell offices, occupants of flex offices were less likely to take any sick leave. [21] The later study reported that men in flex offices were more likely to take two or more short sickness absence episodes compared to men in cell offices. [20] Therefore, in this study, we attempt to reproduce these earlier, inconsistent findings in an independent sample.

**Table 1:** Summary of previous research examining associations between office type and sickness absence.

Study	Outcome(s)	Exposure(s)	Sample	Results
Bodin Danielsson & Bodin, 2008	Over previous 12 months: 1. Any sick leave (self-reported) 2. More than 7 days of sick leave (self-reported).	Office type: Cell (1 occupant), shared (2–3), small open-plan (4–9), medium open-plan (10–24), large open-plan (>24), flex, combi.	469 employees from a convenience sample of offices in Stockholm, Sweden.	Occupants of flex offices less likely to take any sick leave in previous year (outcome 1). No other relationships significant at 95% level.
Pejtersen et al., 2011	Over previous 12 months: Number of days of sickness absence (self-reported).	Office type (number of occupants): 1, 2, 3–6, >6.	2403 Danish employees responding to a national survey.	More days of sickness absence reported by employees in shared or open-plan offices than employees working in cell offices.
Bodin Danielsson et al., 2014	Over previous 12 months: 1. Two or more short sick leave spells of one week or less (self-reported) 2. Any long (medically certified) sickness absence spells over one week long (self-reported) 3. Whether reported more than 7 days of sick leave in total (self-reported).	Office type: Cell (1 occupant), shared (2–3), small open-plan (4–9), medium open-plan (10–24), large open-plan (>24), flex, combi.	1852 Swedish employees responding to a national survey.	Compared to employees in cell offices: All employees and female employees more likely to report two or more short sick leave spells of one week or less if working in small, medium or large open-plan offices (outcome 1). Female employees more likely to report a long sickness absence spell if working in large open-plan offices (outcome 2). Male employees more likely to report short sick leave spells and more than 7 days of sick leave in total if working in flex offices (outcomes 1 & 3). No other relationships significant at 95% level.

The second way in which we aim to contribute new knowledge is in using a more robust study design than previous work. An important limitation concerning all earlier findings is that they rely on self-reported sickness absence, which may be affected by incorrect recall. For this reason, the current study additionally includes employer records of sickness absence, thereby ruling out the possibility that recall bias has affected the findings.

Lastly, this study includes sickness presence as an outcome, since we are not aware of any previous studies examining the relationship between office type and sickness presence. Understanding this relationship would cast light on the mechanisms underlying sickness absence, by indicating whether office type might affect sickness absence as a result of its impact on vulnerability to ill health (if sickness absence and presence are positively correlated and have a similar relationship with office type) or upon the decision-making process to attend work (if sickness absence and presence have opposite relationships with office type).

In short, the aim of the present study is to investigate the relationship between office type and both sickness absence and presence, taking an approach which complements self-reports of sickness absence with employer records. We hypothesize that employees in office types containing more workers will report more days and episodes of sickness absence as well as more days of presence, in a graded manner such that participants in cell offices will report least sickness absence and presence and those in open-plan offices the most. Since flex offices can vary greatly in density and previous findings have been inconsistent, we do not generate hypotheses in relation to flex offices. Since one study has indicated differences in effects by gender, we will examine gender interactions in our analyses.

## 2. Materials and methods

### 2.1. Study design and sample

This study employed a cross-sectional design to investigate the impact of office type on employer records and self-reports of sickness absence as well as self-reports of presence. In a hierarchical sampling strategy, recruitment to the study was first carried out at the organizational level and then at the level of departments and office buildings. In order to make data collection time and cost-effective, organizations were eligible for inclusion if they had office buildings containing at least 50 employees per building located in the same city as the research team or had office buildings located elsewhere with at least 100 employees per building. Another inclusion criterion was that the organization should use different types of offices, e.g., both cell offices and open-plan offices. Ten eligible organizations were found and their human resources or facility managers were contacted to seek approval for the project in the managerial board and with trade unions. Five organizations with suitable office buildings agreed to take part in the study, of which three provided employee absence data. Two organizations are in the private sector: one developing and supporting high tech products and the other working in building construction; the third organization, in the public sector, is a work placement service.

After approval was given, departments and office buildings with various office designs were selected in order to include a sufficient number of employees in each studied office type within these organizations. An exception was made to include a division within the public sector organization with about 500 employees organized in minor groups scattered in various locations, not all visited by the researchers. Departments that were in a change process regarding the physical design of the office were not included in the study. Office managers were asked if their department could participate and most accepted the invitation.

Employees completed an e-survey (response rate: 69.5%) and personnel departments provided absence data, which contained information about leave due to sickness as well as other absences such as vacation or parental leave. The e-survey could be answered during a one-month period and was collected during January–early July 2012.

Across Sweden, 2859 professionals or higher grade clerks were recruited to the study. Several groups were excluded to leave a total of 2078 eligible participants: employees spending <50% of their working time at the office or <25% of their working time at their work stations; employees who had been working < 3 months at their current workplace and employees who worked in their own room where others in their unit were in shared spaces, because this might have been for health reasons.

Because of missing data, two separate samples were created. The sample using employer records of sickness absence contained 988 participants, after exclusions for 396 participants lacking employer absence records, 114 people who joined or left the employer during the year in which sickness absences were recorded, 115 people who were away for more than five months on non-sick leave and people with missing information on education (n = 396) or office type (n = 67). The sample using self-reports of sickness absence and presence contained 1237 participants, after 841 participants with missing data on one or more covariates were excluded.

The Regional Ethical Review Board in Stockholm approved the study. The study has been conducted in accordance with the American Psychology Association’s ethical standards.

### 2.2. Measures

#### Outcomes

Number of days and number of episodes of sickness absence were obtained from employer records; participants provided self-reports of their levels of sickness absence and presence in the e-survey.

#### Employer sickness absence records

Each employer’s human resources department provided absence records, including sickness absence, for a 12-month period which, depending on the organization, took place during October/November 2011–September/October 2012. Because it is valuable to distinguish lengthy sickness absence spells from regular short spells, both the number of days and number of episodes of sickness absence were totalled for each employee. Distinct episodes of sickness absence were separated by returns of at least one day to the workplace.

#### Self-reported sickness absence

Employees from all three organizations responded to the e-survey question: “For roughly how many days in total have you been on sick leave during the past 12 months?” which had the following response categories: None, 1–3 days, 4–7 days, 8–14 days, 15–30 days, 31–90 days, 91 days or more. Since the e-survey data were collected in January–early July 2012, this 12-month period of retrospective recall overlapped only partially with employer sickness absence records which were collected over the period October/November 2011–September/October 2012.

#### Self-reported sickness presence

A question on sickness presence was included in the e-survey: “For roughly how many days in total have you gone to work knowing that, owing to your condition, you ought to have reported in sick in the past 12 months?” which also had the response categories: None, 1–3 days, 4–7 days, 8–14 days, 15–30 days, 31–90 days, 91 days or more.

*Exposure*. *Office type*: Recent research distinguishes cell offices, shared offices, open-plan offices of varying sizes and flex offices in which employees lack a fixed workstation. [20,22–24] Therefore, locations of participants’ workstations were categorized into six office types: 1. cell offices containing one workstation, 2. shared offices containing 2–3 workstations, 3. small open-plan offices with 4–9 workstations, 4. medium-sized open-plan offices containing 10–24 workstations, 5. large open-plan offices with more than 24 workstations and 6. flex offices.

During visits to the office buildings, the second author classified participants’ workstations by plotting their locations on architectural drawings provided by the organizations. This approach ensured consistency across office buildings and provided the researcher with complete information about the nature of the offices (e.g., that there were no cubicle designs where screens or glass walls reach almost to the ceiling). Within the public sector organization, around 500 employees were organized in minor groups scattered in various locations and the researchers could not visit about 330 of these workstations. These workstations were categorized from respondents’ responses to the e-survey in response to three questions: “At your workplace, do you have your own room or do you share your room with others?” “At your workplace, do you have your own workstation?” “If you share your room with others, stand up and count how many workstations you see from your own workstation.” If a respondent responded that they did not have their own workstation, they were categorized into a flex office.

For 996 participants, both researcher and e-survey responses were available: these corresponded in 76% of cases. Most of the mismatched cases were due to assessments differing by either one size smaller or larger open-plan office type (88%); in 46 cases (12%), the disagreement was more substantial. Where there was lack of agreement, the researcher’s classification was used.

#### Covariates

The covariates included in the model were gender (0 = male, 1 = female), age (continuous, 20–68 years), educational level (0 = no academic degree, 1 = an academic degree) and labour market sector (0 = public, 1 = private).

### 2.3. Data analysis

Since the count data of number of days absent due to sickness and number of episodes of sickness absence obtained from employer records had overdispersed distributions (in which the variance is greater than the mean, Table 2), they were analysed using negative binomial regression. The self-reported measures of sickness absence and presence were modelled using ordinal logistic regression. For each outcome, two models were run on complete cases, the first to obtain unadjusted results for office type and each control variable, and a second mutually adjusted model. All analyses were performed in Stata 15.1.

**Table 2:** Description of the respondents in the employer records of sickness absence sample (n = 988).

Office type	n	Female sex %	Mean age in years (SD)	Educational level (high) %	Private sector %	Mean sick leave days (SD)	Mean sick leave episodes (SD)
Cell	212	71.1	49.4 (9.9)	79.7	20.3	12.0 (33.2)	1.7 (2.1)
Shared	74	64.9	47.0 (10.1)	89.2	6.8	7.7 (15.8)	1.6 (1.5)
Small open-plan	146	69.2	45.9 (10.6)	69.2	15.1	9.0 (24.4)	1.5 (1.9)
Medium-sized open-plan	278	56.8	48.6 (10.5)	70.1	29.1	8.7 (21.3)	1.6 (2.1)
Large open-plan	240	54.2	48.3 (10.7)	72.5	28.3	9.4 (26.6)	1.4 (1.7)
Flex	38	36.8	51.8 (11.1)	84.2	18.4	21.9 (77.6)	1.3 (1.8)
Total	988	60.8	48.3 (10.5)	74.6	22.9	10.1 (27.4)	1.5 (1.9)

We performed two sensitivity analyses. Because previous research has suggested gendered relationships between office type and sickness absence, [20] in a sensitivity analysis we introduced interactions between gender and office type for each of the outcomes, performing F-tests and examining coefficients. In a second sensitivity analysis, we modelled the company records of sickness absence (days and episodes of sick leave) after multiple imputation of missing values for the education variable (n = 1374 after multiple imputation, 40 imputations).

## 3. Results

Participants worked in a range of office types, predominantly in cell and open-plan offices, while flex offices were the rarest type (Tables 2 and 3). In both samples, most employees were female, educated to degree level and worked in the public sector. Their average age was around 48 years.

**Table 3:** Description of the respondents in the self-reported sickness absence and sickness presence sample (n = 1237).

Office type	n	Female sex %	Mean age in years (SD)	Educational level (high) %	Private sector %	Days of sickness absence (%)				Days of sickness presence (%)			
						0	1–3	4–7	≥8	0	1–3	4–7	≥8
Cell	283	70.3	49.4 (10.5)	80.9	20.9	32.9	27.9	21.9	17.3	30.0	38.2	17.0	14.8
Shared	99	72.7	46.0 (9.8)	87.9	7.1	24.2	32.3	21.2	22.2	27.3	42.4	14.1	16.2
Small open-plan	204	72.1	45.7 (10.7)	70.1	18.1	29.4	30.4	20.6	19.6	31.9	40.7	14.7	12.8
Medium-sized open-plan	339	57.5	48.2 (10.4)	69.6	32.7	27.7	26.0	21.5	24.8	29.5	39.2	18.6	12.7
Large open-plan	274	54.0	47.8 (10.8)	70.8	32.5	31.0	31.0	18.3	19.7	35.4	34.3	17.9	12.4
Flex	38	52.6	50.2 (10.6)	84.2	18.4	39.5	21.1	15.8	23.7	42.1	39.5	13.2	5.3
Total	1237	63.1	47.8 (10.6)	74.5	25.1	30.0	28.6	20.5	20.9	31.5	38.4	16.9	13.2

According to employer records, mean annual number of sick leave days was 10.1, but varied between office types from 7.7 days for employees in shared offices to 21.9 days for employees in flex offices (Table 2). Employees were absent for a mean number of 1.5 episodes of sick leave; there was relatively little variation around this number.

Rates of self-reported sickness absence and presence appeared to vary between office types. On average, 30% of employees reported taking no sickness absence and 32% reported no sickness presence during the previous 12 months. There were variations across office types, but few clear trends are apparent.

**Fig 1:**
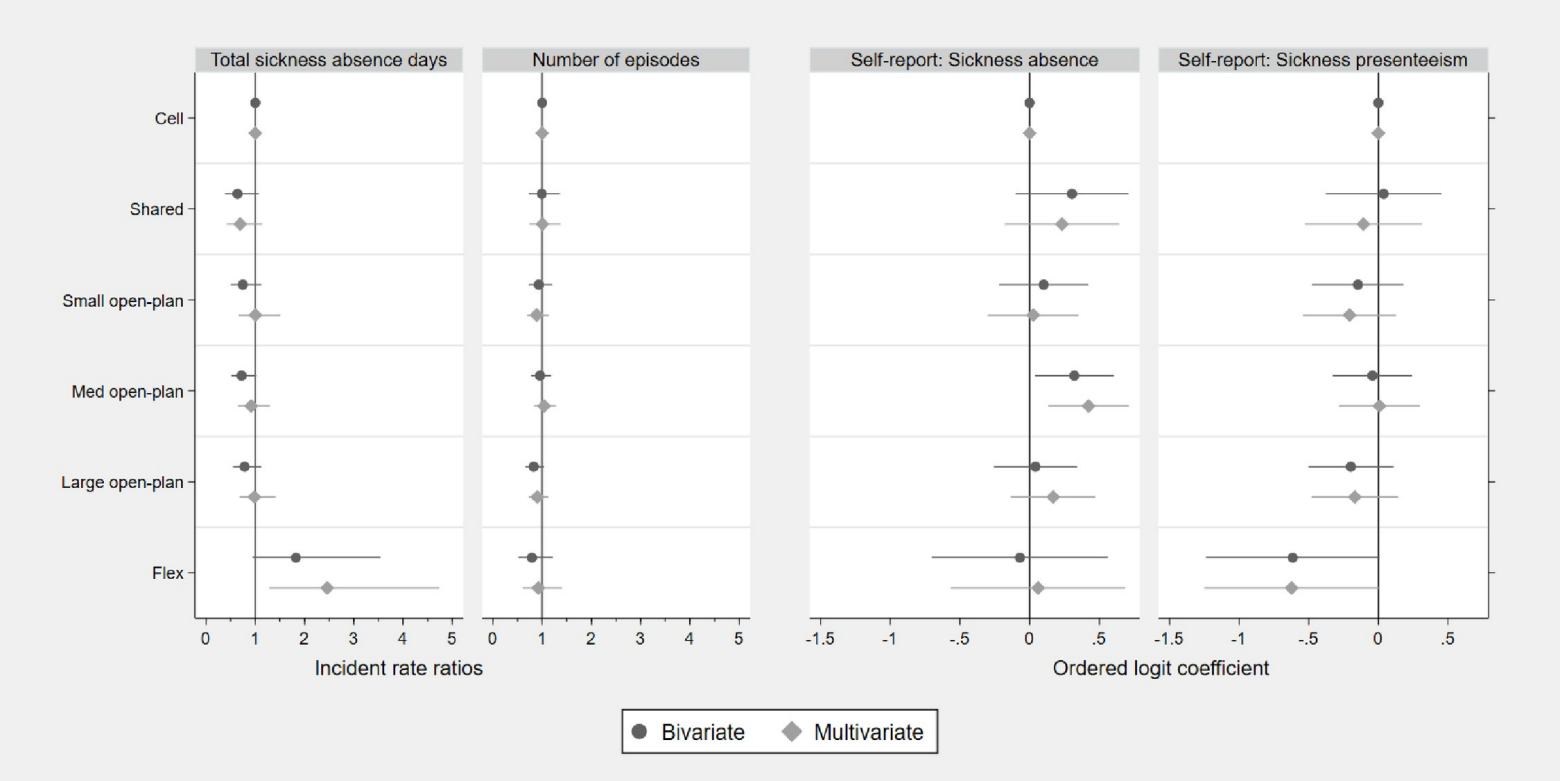
Associations between office type and employer records of days of sickness absence and number of episodes of sickness absence over the previous 12 months, *n* = 988, as well as self-reported days of sickness absence and sickness presence over the previous 12 months, *n* = 1237. Error bars indicate 95% confidence intervals. Multivariate estimates were generated after adjusting for gender, age, education level and public or private sector. Office types: Cell (1 workstation), shared (2–3 workstations), small open-plan (4–9 workstations), medium open-plan (10–24 workstations), large open-plan (over 24 workstations), flex (no allocated workstation).

Correlations between the main covariates (gender, age, education level and sector) and outcomes are displayed in Tables 4 and 5. Office type was regressed on employer records of sickness absence using negative binomial regressions, expressed in incident rate ratios. In the unadjusted results, women, older participants, those with academic degrees and public sector participants had more days absent than men, those without academic degrees and younger participants and private sector workers, respectively (Table 6, Fig 1). Women, younger and private sector workers had more episodes of sick leave. In the unadjusted results, no office type was significantly different to cell offices, at the 5% significance level, in terms of either number of days or episodes of absence.

**Table 4:** Correlations between the main covariates in the employer records of sickness absence sample (n = 988).

	1.	2.	3.	4.	5.	6.
1. Gender	1					
2. Age	–0.06	1				
3. Education level	–0.01	–0.17***	1			
4. Sector	–0.19***	–0.08*	–0.08*	1		
5. Days of sickness absence (employer records)	0.10**	0.08*	0.05	–0.07*	1	
6. Episodes of sickness absence (employer records)	0.17***	–0.11***	–0.05	–0.13***	0.31***	1

**Table 5:** Correlations between the main covariates in the self-reported sickness absence and sickness presence sample (n = 1237).

	1.	2.	3.	4.	5.	6.
1. Gender	1					
2. Age	–0.09**	1				
3. Education level	0.00	–0.19***	1			
4. Sector	–0.19***	–0.08*	–0.13***	1		
5. Sickness absence (self-report)	0.18***	–0.03	–0.04	–0.10***	1	
6. Sickness presence (self-report)	0.04	–0.08*	0.07*	–0.12***	0.32***	1

**Table 6:** Associations of office type with employer records of sickness absence: Results from negative binomial regressions (n = 988).

	Days of sickness absence (employer records)		Episodes of sickness absence (employer records)	
Variable	Unadjusted incident rate ratios (95% CI)	Fully adjusted incident rate ratios (95% CI)	Unadjusted incident rate ratios (95% CI)	Fully adjusted incident rate ratios (95% CI)
Gender				
Male (ref.)	1	1	1	1
Female	1.83*** (1.43; 2.34)	1.82*** (1.42; 2.35)	1.58*** (1.35; 1.85)	1.46*** (1.25; 1.72)
Age	1.02** (1.01; 1.03)	1.02*** (1.01; 1.03)	0.99** (0.98; 0.99)	0.99*** (0.98; 0.99)
Education level				
No academic degree (ref.)	1	1	1	1
Academic degree	1.39* (1.05; 1.84)	1.40* (1.06; 1.84)	0.88 (0.74; 1.04)	0.81* (0.68; 0.96)
Sector				
Public (ref.)	1	1	1	1
Private	0.60** (0.45; 0.80)	0.72* (0.53; 0.98)	0.65*** (0.54; 0.79)	0.69*** (0.57; 0.84)
Office type				
Cell (ref.)	1	1	1	1
Shared	0.64 (0.38; 1.07)	0.69 (0.42; 1.14)	0.99 (0.72; 1.36)	1.07 (0.74; 1.37)
Small open-plan	0.75 (0.50; 1.12)	1.00 (0.67; 1.51)	0.93 (0.72; 1.20)	0.89 (0.65; 1.14)
Medium open-plan	0.72 (0.51; 1.02)	0.92 (0.65; 1.29)	0.96 (0.77; 1.19)	1.04 (0.84; 1.28)
Large open-plan	0.78 (0.55; 1.12)	0.98 (0.68; 1.41)	0.82 (0.66; 1.04)	0.90 (0.72; 1.12)
Flex	1.83 (0.94; 3.54)	2.46** (1.28; 4.73)	0.79 (0.52; 1.22)	0.92 (0.61; 1.41)

* p<0.05

** p<0.01

***p<0.001

After mutual adjustment for all of the covariates, the control variables apart from sector were all associated with number of days absent. Turning to office type, the only difference was the greater number of days absent in flex offices compared to cell offices (IRR = 2.46, p = 0.007). Gender, age and public/private sector were correlated with number of episodes of absence, not office type.

Office type was regressed on self-reported sickness absence and sickness presence using ordered logistic regression, expressed in log-odds coefficients. In unadjusted analyses, higher rates of self-reported sickness absence were associated with female gender, younger age and working in the public sector (Table 7). The only difference from the odds in cell offices significant at the 5% level was in the medium open-plan offices (0.32, p = 0.026) (Fig 1). Broadly similar results were obtained after performing mutual adjustment. In particular, participants working in medium open-plan offices had 0.42 higher log-odds of self-reported sickness absence than those working in cell offices (p = 0.004).

**Table 7:** Associations of office type with self-reported sickness absence and self-reported sickness presence: Results from ordered logistic regressions (n = 1237).

	Self-reported sickness absence		Self-reported sickness presence	
Variable	Unadjusted ordered logit coefficient (95% CI)	Fully adjusted ordered logit coefficient (95% CI)	Unadjusted ordered logit coefficient (95% CI)	Fully adjusted ordered logit coefficient (95% CI)
Gender				
Male (ref.)	0	0	0	0
Female	0.73*** (0.53; 0.95)	0.70*** (0.48; 0.92)	0.11 (–0.10; 0.32)	–0.02 (–0.24; 0.20)
Age	–0.01* (–0.02; –0.00)	–0.01* (–0.02; –0.00)	–0.02*** (–0.03; –0.01)	–0.02*** (–0.03; –0.01)
Education level				
No academic degree (ref.)	0	0	0	0
Academic degree	–0.14 (–0.37; 0.09)	–0.21 (–0.46; 0.03)	0.30* (0.07; 0.54)	0.16 (–0.09; 0.41)
Sector				
Public (ref.)	0	0	0	0
Private	–0.36** (–0.59; –0.13)	–0.30* (–0.54; –0.06)	–0.43*** (–0.66; –0.20)	–0.47*** (–0.72; –0.23)
Office type				
Cell (ref.)	0	0	0	0
Shared	0.31 (–0.10; 0.71)	0.23 (–0.18; 0.64)	0.04 (–0.38; 0.45)	–0.11 (–0.53; 0.31)
Small open-plan	0.10 (–0.21; 0.42)	0.03 (–0.30; 0.35)	–0.15 (–0.48; 0.18)	–0.21 (–0.54; 0.13)
Medium open-plan	0.32* (0.04; 0.61)	0.42** (0.13; 0.71)	–0.04 (–0.33; 0.24)	0.01 (–0.28; 0.30)
Large open-plan	0.04 (–0.25; 0.34)	0.17 (–0.13; 0.47)	–0.20 (–0.50; 0.11)	–0.17 (–0.48; 0.14)
Flex	–0.07 (–0.70; 0.56)	0.06 (–0.56; 0.69)	–0.61 (–1.24; 0.01)	–0.62 (–1.25; 0.00)

* p<0.05

** p<0.01

***p<0.001

Higher log-odds of self-reported sickness presence were associated with younger age, having an academic degree and working in the public sector in unadjusted analyses, but not with office type. After mutual adjustment, only age and sector were significantly associated with sickness presence.

We performed two sensitivity analyses. In order to examine whether the genders might differ in the associations between office type and sickness absence and presence, we examined interactions between gender and office type for each of the four outcomes. In each case, the results for F-tests indicated that addition of interactions did not improve model fit at the 5% significance level, and therefore we do not report the interactions.

In the second sensitivity analysis, missing values for education were imputed which increased the sample size for the employer records from 988 to 1374. Results obtained were broadly similar to those in the main analysis (S1 and S2 Tables). Small differences were that those working in medium open-plan offices had fewer days of sickness absence, while those working in large open-plan offices had fewer episodes of sickness absence, findings which did not reach the 5% level of significance in the fully adjusted model.

Although prior research distinguishes different office sizes, we observed similarities in the results between the different types of multi-person office. A reviewer suggested conducting post-hoc analyses comparing cell offices with all other office types together. In both bivariate and fully adjusted models, there were no differences at the 95% significance level in company-recorded sickness absence days (full adjustment: p = 0.974) or episodes (p = 0.654), or in self-reported sickness absence days (p = 0.064) or sickness presence days (p = 0.318) compared to working in cell offices.

## 4 Discussion

The overall aim of the present study was to investigate the relationship of office type with sickness absence and presenteeism. It examined whether employees working in offices containing more co-workers would report greater sickness absence and presence, with four outcomes using both self-reported and employer data and examining open-office types ranging from small to large.

Associations between both self-reported and employer-recorded sickness absence with gender and employer sector were as expected. [25,26] However, this study failed to confirm the hypothesis that, compared to cell offices, open-plan offices are associated with higher rates of sickness absence and presence, despite testing many comparisons: four outcomes, bivariate and multivariate associations, two specifications of office type (cell offices vs each of five other office types; cell offices vs all other office types). Consequently, the single association found in support of this hypothesis, between working in a medium-sized open plan office and higher self-reported sickness absence, is most likely due to chance. After imputation of missing values for education, the employer-recorded sickness absence sample size increased to 1374 participants, but the results provided no indication that open-plan offices were associated with more days or episodes of sickness absence. This generally negative set of findings for sickness absence corresponds to the report by Bodin Danielsson & Bodin (2008) but is in contrast to the reports from Pejtersen et al. (2011) and Bodin Danielsson et al. (2014). [19–21]

The finding that flex offices were associated with more days of employer-recorded sickness absence does correspond to a previous report of higher rates of self-reported sickness absence for male employees in flex offices, although we were not able to reproduce the earlier report with the self-reported sickness absence measure. [20] In light of the ongoing trend for conversion of traditional and open-plan offices into activity-based flex offices, further research is needed into the nature of the work environment in such offices and any effect on sickness absence. [1]

### 4.1. Possible explanations for the findings

Some alternative explanations for the negative findings concerning open-plan offices in general are that the effects upon sickness absence and presence of office type may be quite small. It may be that, compared to other work environment factors, such as physical and psychosocial occupational exposures, quality of leadership or work-family conflict, office type exerts a minor effect, if any, upon sickness absence and presenteeism. Additionally, any effects may depend on a causal chain of intermediary factors, the time lag for which is unknown. Such a possibility is strengthened by results from previous work which found that office type was associated with immediate outcomes such as distraction and cognitive stress, but not with more downstream health outcomes. [11]

It is also possible that effects in both directions occur which cancel each other out. For example, there may be greater social pressure in open-plan offices not to take sickness absence since absences may be more easily noticed by others (attendance requirements), but employees in open-plan offices may have reduced adjustment latitude in terms of making their work environment more comfortable by reducing noise and other distractions.

Finally, the results may be confounded by a third variable which was not included. People were not randomly allocated to the different office types and it may be that office type is associated with the sort of role a person has, and therefore with their psychosocial working conditions more generally. In addition, individual strategies and responses may plan an important role. Participants may have adapted to open-plan working in a variety of ways, such as by wearing earphones, learning to screen out distraction, and performing more concentrated tasks at less busy times. Another aspect that may explain our null findings is that since the first day of sickness absence is not paid in Sweden and a reduced salary is paid on other sick days, individuals may choose to take better reimbursed forms of leave, such as holiday or child-care leave, or to work from home, rather than take sickness absence. [27] There may be also selection processes in which employees who feel that their working conditions are harming their health and well-being seek employment in a different environment.

### 4.2. Strengths and limitations

This is the first study to examine the association of office type with both self-reported and employer records of sickness absence and, in addition, examines the relationship with subjective assessments of sickness presence for the first time. Use of self-reported sickness absence days allows these findings to be compared with earlier studies using similar measures. By additionally including employer data on sickness absence, we could exclude the possibility that findings might have been affected by participants recalling their absences due to illness inaccurately. In addition to days of absence, by counting episodes of absence we were able to allow for the possibility that office type might have generated repeated occurrences of short spells of sickness absence as opposed to few spells of longer duration. A high frequency of absences, even if they are short-term absences, can be more disruptive and difficult for employers to plan for. An additional strength of the study is that, in most cases, classification of office type was based on both participant self-reports and external judgment by one of the co-authors. Participants who spent little time in their offices, or who had begun working in the office or organization for fewer than three months before responding to the e-survey were excluded.

However, the study does have certain limitations. The organizations included in the study were representative of the public and private sector, but the generalizability of the study is limited to large employers in Sweden. The sample size is such that it would not have been possible to observe small effects, although it is sufficiently powered to find effects of the size reported in previous research. An important limitation is that companies aware that they had problems with their office environments would not have been willing to participate. The effect of office type on sickness absence may be smaller in organizations that have well-functioning office space, providing a quiet environment or a relatively high degree of visual privacy.

## Conclusions

We were unable to demonstrate that open-plan offices were associated with higher rates of sickness absence than individual offices, nor with higher rates of sickness presence. While there may be gains in productivity and well-being from better workplace design, it appears that factors other than office type may be determining rates of sickness absence.

## Supporting information

S1 Table Associations of office type with employer records of sickness absence: Results from negative binomial regressions following multiple imputation of missing education data (n = 1374). (DOCX)

S2 Table Associations of office type with employer records of sickness absence: Results from negative binomial regressions (n = 988). (DOCX)
