# Supplementary material for: Sickness absence and sickness presence in relation to office type: An observational study of employer-recorded and self-reported data from Sweden
Source: PLoS One. 2020 Apr 29;15(4):e0231934. doi: 10.1371/journal.pone.0231934 (PMC7190108; doi:10.1371/journal.pone.0231934)
Supplement: S1 Table — (DOCX) [file pone.0231934.s001.docx]

Table S1: Associations of office type with employer records of sickness absence: Results from negative binomial regressions following multiple imputation of missing education data (n=1374).

|  | **Days of sickness absence (employer records)** | | **Episodes of sickness absence (employer records)** | |
| --- | --- | --- | --- | --- |
| **Variable** | **Unadjusted coefficients (95% CI)** | **Fully adjusted coefficients (95% CI)** | **Unadjusted coefficients (95% CI)** | **Fully adjusted coefficients (95% CI)** |
| Gender |  |  |  |  |
| Male (ref.) | 0 | 0 | 0 | 0 |
| Female | 0.61*** (0.40; 0.82) | 0.56*** (0.34; 0.78) | 0.45*** (0.31; 0.59) | 0.35*** (0.21; 0.49) |
| Age | 0.02*** (0.01; 0.03) | 0.03*** (0.01; 0.03) | –0.01** (–0.02; –0.00) | –0.01*** (–0.02; –0.01) |
| Education level |  |  |  |  |
| No academic degree (ref.) | 0 | 0 | 0 | 0 |
| Academic degree | 0.35* (0.08; 0.62) | 0.37* (0.05; 0.60) | –0.18 (–0.37; 0.01) | –0.26** (–0.45; –0.07) |
| Sector |  |  |  |  |
| Public (ref.) | 0 | 0 | 0 | 0 |
| Private | –0.75*** (–1.00; –0.51) | –0.48*** (–0.79; –0.28) | –0.52*** (–0.68; –0.35) | –0.47*** (–0.63; –0.30) |
| Office type |  |  |  |  |
| Cell (ref.) | 0 | 0 | 0 | 0 |
| Shared | –0.10 (–0.56; 0.37) | –0.23 (–0.64; 0.26) | –0.11 (–0.41; 0.18) | –0.13 (–0.42; 0.15) |
| Small open-plan | –0.29 (–0.66; 0.08) | 0.08 (–0.36; 0.38) | –0.07 (–0.30; 0.16) | –0.15 (–0.37; 0.08) |
| Medium open-plan | –0.35* (–0.65; –0.04) | –0.18 (–0.43; 0.18) | –0.10 (–0.29; 0.09) | 0.03 (–0.21; 0.16) |
| Large open-plan | –0.29 (–0.60; 0.03) | –0.07 (–0.38; 0.24) | –0.20* (–0.40; –0.00) | –0.13 (–0.32; 0.06) |
| Flex | 0.64 (–0.03; 1.32) | 0.96** (0.25; 1.57) | –0.21 (–0.65; 0.23) | –0.09 (–0.52; 0.34) |

** p<0.05, ** p<0.01, ***p<0.001*
