# Supplementary material for: Sickness absence and sickness presence in relation to office type: An observational study of employer-recorded and self-reported data from Sweden
Source: PLoS One. 2020 Apr 29;15(4):e0231934. doi: 10.1371/journal.pone.0231934 (PMC7190108; doi:10.1371/journal.pone.0231934)
Supplement: S2 Table — (DOCX) [file pone.0231934.s002.docx]

Table S2: Associations of office type with employer records of sickness absence: Results from negative binomial regressions (n=988).

|  | **Days of sickness absence (employer records)** | | **Episodes of sickness absence (employer records)** | |
| --- | --- | --- | --- | --- |
| **Variable** | **Unadjusted coefficients (95% CI)** | **Fully adjusted coefficients (95% CI)** | **Unadjusted coefficients (95% CI)** | **Fully adjusted coefficients (95% CI)** |
| Gender |  |  |  |  |
| Male (ref.) | 0 | 0 | 0 | 0 |
| Female | 0.60*** (0.36; 0.85) | 0.60*** (0.35; 0.85) | 0.46*** (0.30; 0.62) | 0.38*** (0.22; 0.54) |
| Age | 0.02** (0.01; 0.03) | 0.02*** (0.01; 0.03) | –0.01** (–0.02; –0.01) | –0.01*** (–0.02; –0.01) |
| Education level |  |  |  |  |
| No academic degree (ref.) | 0 | 0 | 0 | 0 |
| Academic degree | 0.33* (0.05; 0.61) | 0.33* (0.05; 0.61) | –0.13 (–0.30; 0.04) | –0.22** (–0.39; –0.05) |
| Sector |  |  |  |  |
| Public (ref.) | 0 | 0 | 0 | 0 |
| Private | –0.51** (–0.79; –0.22) | –0.33* (–0.63; –0.02) | –0.43*** (–0.62; –0.24) | –0.37*** (–0.57; –0.18) |
| Office type |  |  |  |  |
| Cell (ref.) | 0 | 0 | 0 | 0 |
| Shared | –0.45 (–0.96; 0.07) | –0.37 (–0.87; 0.14) | –0.01 (–0.32; 0.31) | –0.01 (–0.30; 0.31) |
| Small open-plan | –0.29 (–0.70; 0.12) | 0.02 (–0.40; 0.41) | –0.07 (–0.32; 0.18) | –0.12 (–0.36; 0.13) |
| Medium open-plan | –0.33 (–0.67; 0.02) | –0.09 (–0.43; 0.26) | –0.04 (–0.26; 0.17) | 0.04 (–0.17; 0.25) |
| Large open-plan | –0.24 (–0.60; 0.11) | –0.02 (–0.38; 0.32) | –0.19 (–0.41; 0.04) | –0.10 (–0.32; 0.12) |
| Flex | 0.60 (–0.06; 1.26) | 0.90** (0.25; 1.55) | –0.23 (–0.66; 0.20) | –0.08 (–0.50; 0.34) |

** p<0.05, ** p<0.01, ***p<0.001*

Note: This table is provided for comparison purposes.
